# Influence of PEG coating on the oral bioavailability of gold nanoparticles in rats

**DOI:** 10.1080/10717544.2017.1282554

**Published:** 2017-02-21

**Authors:** Ahmed Alalaiwe, Georgia Roberts, Paul Carpinone, John Munson, Stephen Roberts

**Affiliations:** 1 Department of Pharmaceutics, College of Pharmacy, University of Florida, Gainesville, FL, USA,; 2 Center for Environmental and Human Toxicology, University of Florida, Gainesville, FL, USA, and; 3 Major Analytical and Particle Analysis Instrumentation Centers, University of Florida, Gainesville, FL, USA

**Keywords:** Gold nanoparticles, oral bioavailability, polyethylene glycol (PEG), metallic nanoparticles, bioavailability assessment

## Abstract

Metallic nanoparticles can be produced in a variety of shapes, sizes, and surface chemistries, making them promising potential tools for drug delivery. Most studies to date have evaluated uptake of metallic nanoparticles from the GI tract with methods that are at best semi-quantitative. This study used the classical method of comparing blood concentration area under the curve (AUC) following intravenous and oral doses to determine the oral bioavailability of 1, 2 and 5 kDa PEG-coated 5 nm gold nanoparticles (AuNPs). Male rats were given a single intravenous dose (0.8 mg/kg) or oral (gavage) dose (8 mg/kg) of a PEG-coated AuNP, and the concentration of gold was measured in blood over time and in tissues (liver, spleen and kidney) at sacrifice. Blood concentrations following oral administration were inversely related to PEG size, and the AUC in blood was significantly greater for the 1 kDa PEG-coated AuNPs than particles coated with 2 or 5 kDa PEG. However, bioavailabilities of all of the particles were very low (< 0.1%). Concentrations in liver, spleen and kidney were similar after the intravenous doses, but kidney showed the highest concentrations after an oral dose. In addition to providing information on the bioavailability of AuNPs coated with PEG in the 1–5 kDa range, this study demonstrates the utility of applying the blood AUC approach to assess the quantitative oral bioavailability of metallic nanoparticles.

## Introduction

Nanomaterials are currently being used in a variety of health-related products, including pharmaceutical agents and medical devices. Their wide-range of potential compositions and physicochemical properties make nanomaterials attractive for a variety of applications ranging from targeted drug and gene delivery to tissue engineering to novel imaging approaches (Salata, 2004). Metallic nanoparticles are among the nanomaterials of greatest interest given the relative ease with which they can be produced in various shapes, sizes, and surface chemistries. The development of metallic nanoparticles for new therapeutic or diagnostic applications requires a working knowledge of their pharmacokinetics as part of the assessment of both their potential efficacy and their safety. Some potential metallic nanomaterial applications, such as imaging contrast enhancers, involve parenteral administration and characterization of the pharmacokinetics after injection is relatively straightforward. Other applications can involve oral administration of the nanomaterial where determining pharmacokinetic properties such as bioavailability is more challenging.

Gold is an example of a metallic nanoparticle with a variety of potential applications in medicine (Daniel et al., 2001; Daniel & Astruc, 2004; Giljohann et al., 2010). Its biocompatibility is well established and particles of different sizes and shapes can be created with precision (Cho et al., 2009). Gold nanoparticles (AuNPs) also have properties that simplify to some extent the study of their pharmacokinetics. They have low dissolution in biological environments, including the gastrointestinal tract (Hinkley et al., 2015), and the background concentrations of gold in tissues are low. As a result, AuNPs can be quantified relatively easily in tissues simply by digesting tissue samples and measuring the gold content, and the high electron density of gold enhances visualization of the nanoparticles in tissues with electron microscopy. These properties make AuNPs an interesting material for study, not only to advance new uses for them in medicine, but also as a model metallic nanoparticle with which to better understand basic principles of nanoparticle oral absorption.

As with other metallic nanoparticles, surface functionalization is an important facet in the development of AuNPs for medical uses, both to impart useful properties to the nanoparticle and to control agglomeration. Several surface ligands have been studied with AuNPs, including polyethylene glycol (PEG). PEG coating can increase biocompatibility by providing lower protein adsorption compared to the uncoated surface, resulting in reduced immune recognition. PEG coating can also assist targeted drug delivery through the attachment of drugs or molecules to functionalized PEGs (Niidome et al., 2006; Larson-Smith & Pozzo, 2012; Geng et al., 2014). For example, galactose-PEG-coated AuNPs of various sizes achieved higher uptake by the liver compared to PEG-coated AuNPs, likely due to accessing galactose receptor-mediated endocytosis by Kupffer cells of the liver (Bergen et al., 2006). PEG coating increases particle resistance to aggregation/agglomeration in biological environments compared to other coating agents, like hexadecyltrimethylammonium bromide (CTAB), leading to longer circulation half-lives. Niidome et al. (2006) reported that 30 min after a single intravenous dose, 54% of the dose of PEG-coated nanorods was found in the blood, while only 10% of the CTAB-coated nanorods remained (Niidome et al., 2006). Several properties of PEG, including chain length and the density of PEG-loading on the particle surface may influence circulation time of PEG-coated nanoparticles in the blood. The effect of different PEG chain lengths on circulation half-life has been previously studied using 5 nm AuNPs (Lipka et al., 2010). Lipka et al. (2010) found that longer chain lengths (e.g. 10 kDa versus 750 Da) resulted in longer circulation time following intravenous administration. PEG coating can also be important in reducing agglomeration of ingested AuNPs within the GI tract. Native (uncoated) AuNPs were found to agglomerate rapidly and extensively when given orally to mice, while PEG-coated AuNPs remained dispersed as primary particles throughout GI transit (Hinkley et al., 2015).

There are few studies investigating the oral absorption of coated or uncoated AuNPs (Hillyer & Albrecht, 2001; Schleh et al., 2012; Smith et al., 2013; Hinkley et al., 2015). All of these studies evaluated AuNP bioavailability with approaches that involved measurement of gold in tissues and/or excreta (urine, feces). To the best of our knowledge, no study has evaluated bioavailability of AuNPs using the classical method of comparing blood concentration area under the curve (AUC) following intravenous and oral doses or provided basic pharmacokinetic parameters such as clearance and half-life following oral administration.

The objective of this study was to examine the influence of PEG chain length on the oral bioavailability of PEG-coated AuNPs in rats, and in the process evaluate the practicality of using blood AUC comparisons for estimation of oral bioavailability of these metallic nanoparticles. Results were compared with tissue concentration measurements at sacrifice, which is a more common method of obtaining bioavailability information for AuNPs.

## Materials and methods

### Synthesis of AuNPs and PEG-coating

Spherical gold particles with a nominal particle size of 5 nm were synthesized by reduction of gold chloride (Frens, 1972). Sodium borohydride was used as the reducing agent. Briefly, a 5 mL quantity of 100 mM sodium borohydride solution was added to 100 mL of a vigorously stirred solution of 0.25 mM gold chloride and 0.25 mM sodium citrate in deionized water. Prior to PEG-coating, the particles were heated to degrade any residual sodium borohydride and then reacted with excess thiol-terminated 1, 2 and 5 kDa PEG. PEG-coated and uncoated particles were washed and concentrated by centrifugation using molecular weight cutoff filters.

### Particle characterization

Particles were characterized with a Hitachi H7000 transmission electron microscope (Hitachi America Ltd., San Bruno, CA), operated at 110 keV, to verify particle size and shape before coating. Uncoated AuNPs were diluted with distilled water before measurements, and then the diluted mixture was dropped on a 200-mesh copper grid with films for examination. Dynamic light scattering (DLS) was used to determine hydrodynamic radius and particle size distribution on a Microtrac Nanotrac (Microtrac, Inc., Montgomeryville, PA). Hydrodynamic radius was measured in water. Zeta potential was measured in water by DLS with a Brookhaven ZetaPlus instrument (Brookhaven Instruments Corp., Holtsville, NY).

### Animals

Male Wistar rats eight-weeks of age (180–220 g) were purchased from Envigo (Indianapolis, IN). All animals were purchased with surgically placed jugular cannulas. Prior to dosing, rats were housed singly in cages with a light/dark cycle of 12 h and controlled temperature (18–26 °C) and humidity (30–70%). The animals had free access to standard food (Teklad Rodent Diet 7912, Envigo, Indianapolis, IN) and water. Animals in the oral gavage study were fasted 12 h prior to dosing and two hours post-gavage. Animals were euthanized via CO_2_ inhalation at either 24 or 48 h following dosing for the intravenous and oral studies, respectively. Death was confirmed by a secondary method, e.g. thoracotomy. These studies were approved by the University of Florida Institutional Animal Care and Use Committee, and all animals were treated humanely according to criteria provided in the NIH **“**Guide for the Care and Use of Laboratory Animals”.

### AuNP administration

Rats (five per treatment group) were given an oral gavage dose (8 mg/kg) of 1, 2 or 5 kDa PEG-coated 5 nm AuNPs in 1 mL of distilled water. Blood (0.3 ml) was collected from the jugular cannula at 0, 0.5, 1, 2, 4, 8, 12, 24 and 48 h post-gavage. Prior to drawing the blood sample, the cannula was flushed with saline. After removal of the sample, the cannula was filled with saline containing heparin (500 U/ml) to maintain patency. At 48 h post-gavage, animals were euthanized as described above and spleen, kidney and liver samples were collected. To calculate the bioavailability of particles in the oral study, rats (three per treatment group) were given an intravenous dose of 1, 2, or 5 kDa PEG-coated 5 nm AuNPs (0.8 mg/kg in 0.4 ml saline) via the jugular cannula. After dosing the cannula was flushed with heparin/saline to ensure delivery of the entire dose. Pilot studies were performed prior to the experiments to ensure that gold particles did not adsorb to the cannula surface and those dosing and sampling from the same jugular cannula did not result in contamination of blood samples taken over time after the dose. Blood samples (0.3 mL) were taken via the jugular cannula at 0, 0.1, 0.5, 1, 2, 4, 8, 12 and 24 h. After each blood sample, an equivalent volume of saline was administered to maintain blood volume. At 24 h post-administration, animals were euthanized as described above and spleen, kidney and liver samples were collected.

### Measurement of gold in blood and tissue

Gold concentrations in blood, liver, spleen and kidney tissue were measured by ICP-MS. Briefly, tissues (0.5 g ± 0.1 g) and blood (0.3 ml) were digested with 1–2 ml of nitric acid in open 16 mm × 150 mm borosilicate tubes at 140 °C. Samples were heated for varying lengths of time until they were fully digested to a transparent colorless liquid. Hydrogen peroxide (300 μL) was then added to each sample, and the sample was evaporated at 130–140 °C to a final volume of 200 μL. Gold was digested by adding 300 μL of aqua regia (3:1, hydrochloric acid:nitric acid), vortexed for 10–15 s, diluted to 6 mL with distilled water, and then filtered using 0.22 μm hydrophilic PTFE membrane filters (13 mm Millex LG filters, Merck, Darmstadt, Germany).

The concentration of gold in blood or tissue samples was quantified using a Thermo Electron X Series II ICP-MS (Thermo Scientific, West Palm Beach, FL), with indium as an internal standard. The analysis was carried out using a six-point standard curve covering six orders of magnitude. A series of gold standard solutions (0.1, 1, 10, 100, 1000 and 10,000 ng/mL) were prepared from a stock standard solution of 1 mg/mL in 2% nitric acid purchased from Fisher Scientific (New Jersey, NJ). The standard curve was obtained from the linear relationship of mean counts per second against a concentration of gold in standard solutions. The lowest standard curve point, 0.1 ng/mL, was used as the limit of quantitation (LOQ). Blanks (2% nitric acid alone) were run in conjunction with all samples. Background gold concentration, measured pre-dose (at T0), was subtracted from measured values to derive the gold concentration attributable to dose. Gold concentration in each sample was determined from the mean of at least five replicate measurements. Tissue concentration values were multiplied by organ weight to estimate total AuNP content in liver, spleen, and kidneys, assuming that the tissue sample was representative of the average concentration within the tissue.

### Pharmacokinetic analysis

Pharmacokinetic parameters from the blood concentration versus time profiles for rats given PEG-coated (1, 2 or 5 kDa) AuNPs were obtained by non-compartmental analysis using Phenix software (Version 2.1 Pharsight, Mountain View, CA). Bioavailability (*F*) was calculated from AUCs following oral and intravenous administration using the following equation.

$$F=\frac{{\mathrm{Dose}}_{\mathrm{iv}}\;\times {\mathrm{AUC}}_{\mathrm{oral}}}{{\mathrm{Dose}}_{\mathrm{oral}}\times \;{\mathrm{AUC}}_{\mathrm{iv}}}$$

### Statistical analysis

Prism 6 (Version: 6.05 GraphPad Software Inc., La Jolla, CA) was used for statistical analysis. Gold (ng/mL) concentrations in blood and tissues are presented as mean ± standard deviation (SD); *N* = 5 for oral doses and *N* = 3 for intravenous doses. Differences among groups were determined using parametric tests. When a significant difference was detected with one-way ANOVA, significant differences among specific groups were identified using a Tukey’s multiple comparisons test. A *p* value** ≤ **0.05 was considered statistically significant.

## Results

### Particle characterization

**Figure 1.**
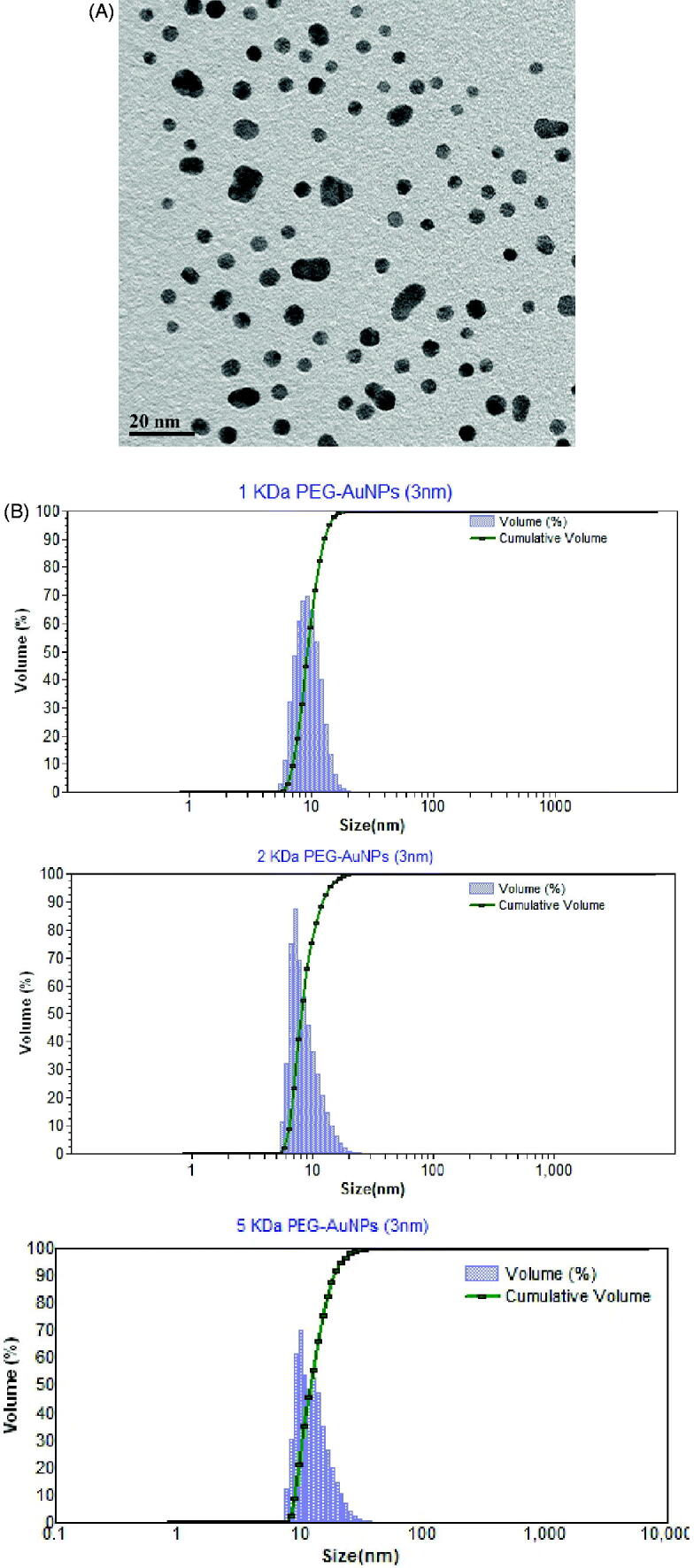
Characterization of AuNPs. Panel A. Transmission electron micrograph of uncoated 5 nm particles. The average primary size was 5 ± 2 nm (mean ± SD) based upon measurement of 100 particles. Panel B. Particle size distribution of 5 nm AuNPs. Particle size distributions for 1 kDa, 2 kDa and 5 kDa PEG-coated particles. The average hydrodynamic radiuses were 12 ± 2, 16 ± 2 and 22 ± 4, respectively (mean ± SD).

AuNPs exhibited an average primary particle size of 5 ± 2 nm (Figure 1). The hydrodynamic radii of the PEG-coated AuNPs were 12 ± 2, 16 ± 2 and 22 ± 4 for 1, 2 and 5 kDa PEG-coated particles, respectively (Figure 1). Zeta potentials for the 1 kDa, 2 kDa and 5 kDa PEG-coated particles in water as administered orally were indistinguishable from neutral: −0.18 ± 0.31, 0.19 ± 0.51 and 1.5 ± 0.9 mV, respectively.

### Pharmacokinetics of PEG-coated AuNPs in blood

**Figure 2.**
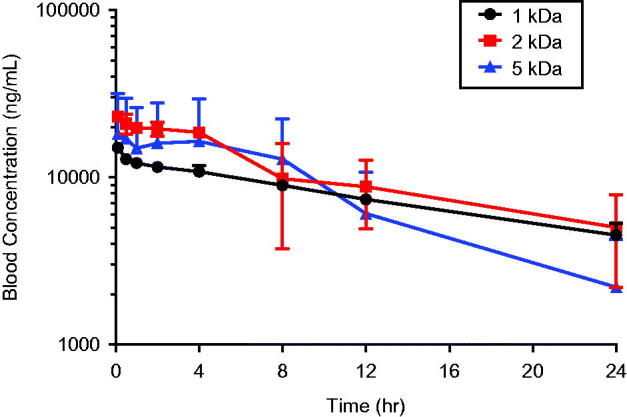
Blood concentration versus time profile following intravenous administration of 1, 2 or 5 kDa PEG-coated 5 nm AuNPs. Rats were administered a single intravenous dose (0.8 mg/kg) of 1, 2 or 5 kDa PEG coated 5 nm AuNPs. Blood samples were taken over time for up to 24 h and AuNP concentrations in blood were measured. Results are plotted beginning with the first sample after the dose (0.1 h) and are expressed as mean ± SD (*N* = 3).

Concentrations of PEG-coated AuNPs administered intravenously (0.8 mg/kg) declined with half-lives ranging from 7 to 17 h (Figure 2, Table 1). Serum elimination half-life appeared to be inversely related to PEG coating size, although the half-lives were not significantly different among treatment groups (*p*** **> 0.05) (Table 1). Clearance, volume of distribution, and AUC were also not significantly different among rats given 1, 2 or 5 kDa PEG-coated particles. The much higher variability in pharmacokinetic parameters in the 5 kDa PEG treated group was due to results from one animal. If that animal is removed from the group, variability in parameters was substantially reduced (T½, 7.1 ± 3.0 h; CL 0.51 ± 0.12 mL/h; Vd, 4.6 ± 0.5 ml; and AUC, 324,600 ± 74,500 h*ng/L), although significant differences among treatment groups were still not observed except for Vd, where the 5 kDa-PEG AuNP treated group has a significantly lower value than either the 1 or 2 kDa-PEG groups.

**Table 1.** Pharmacokinetic parameters following intravenous and oral administration of 1, 2 and 5 kDa PEG-coated gold nanoparticles.

	PEG-coated gold nanoparticle		
Parameter	1 kDa	2 kDa	5 kDa
Intravenous administration (0.8 mg/kg)			
T½ (h)	17.4 ± 2.8	13.2 ± 7.2	7.3 ± 2.0
CL (mL/h)	0.55 ± 0.09	0.54 ± 0.32	1.91 ± 2.44
Vd (mL)	12.6 ± 0.7	8.2 ± 0.9	22.8 ± 31.6
AUC_0 to infinity_ (h*ng/L)	296,700 ± 49,200	364,800 ± 174,500	227,700 ± 175,900
Oral administration (8 mg/kg)			
AUC_0 to infinity_ (h*ng/L)	3,000 ± 1,510^A^	858 ± 170^B^	263 ± 126^B^
*F*	0.0010	0.00023	0.00035

Results are expressed as mean ± SD; *N* = 3 for intravenous doses and *N* = 5 for oral doses.

T ½: half-life; CL: clearance; Vd: volume of distribution; AUC: area under the blood concentration versus time curve; F: bioavailability.

Blood concentration data were analyzed using Phenix software using a non-compartmental approach. Different letter superscripts indicate significant differences among groups, *p* < 0.05.

**Figure 3.**
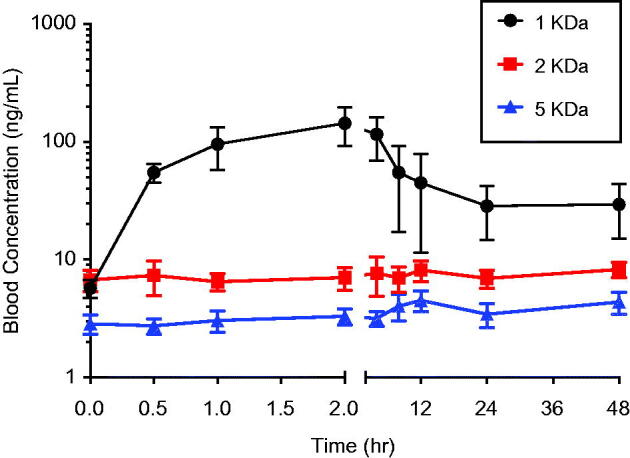
Blood concentration versus time profile following oral administration of uncoated or 1, 2 or 5 kDa PEG-coated 5 nm AuNPs. Rats were administered a single oral dose (8.0 mg/kg) of 1, 2 or 5 kDa PEG coated 5 nm AuNPs by gavage. Blood samples were taken over time for up to 48 hours and AuNP concentrations in blood were measured. Results are plotted beginning with 0 h (just prior to the dose) and are expressed as mean ± SD (*N* = 5).

Anticipating low bioavailability of the AuNPs following oral administration, a 10-fold higher particle dose was administered (8 mg/kg given orally versus 0.8 mg/kg given intravenously). Blood concentrations of gold measured just prior to the oral dose (0 h) reflect background concentrations of gold in blood. Oral administration of the 1 kDa PEG-coated particles resulted in an absorption phase over the first two hours after the dose during which blood concentrations increased, followed by an elimination phase (Figure 3). A discernable absorption phase was not observed for the larger 2 and 5 kDa PEG coated particles, i.e. concentrations of gold in blood appeared indistinguishable from background. Consistent with this, AUCs from 1 kDa PEG coated particle group were significantly higher than those from animals administered 2 or 5 kDa-PEG AuNPs (Table 1). Because this was not a cross-over design experiment in which each animal receives both the oral and intravenous dose of a particle, the oral bioavailabilities of the PEG-coated particles were calculated by comparing the average AUC from animals receiving the oral dose with the average AUC from animals receiving the intravenous dose of the particle (Table 1). The oral bioavailability for all of the PEG-coated particles were ≤ 0.001 (0.1%). Oral bioavailability of the 1 kDa particles was highest, and the bioavailabilities of the 2 and 5 kDa particles were approximately three- to four-fold lower.

### Tissue distribution of PEG-AuNPs

**Figure 4.**
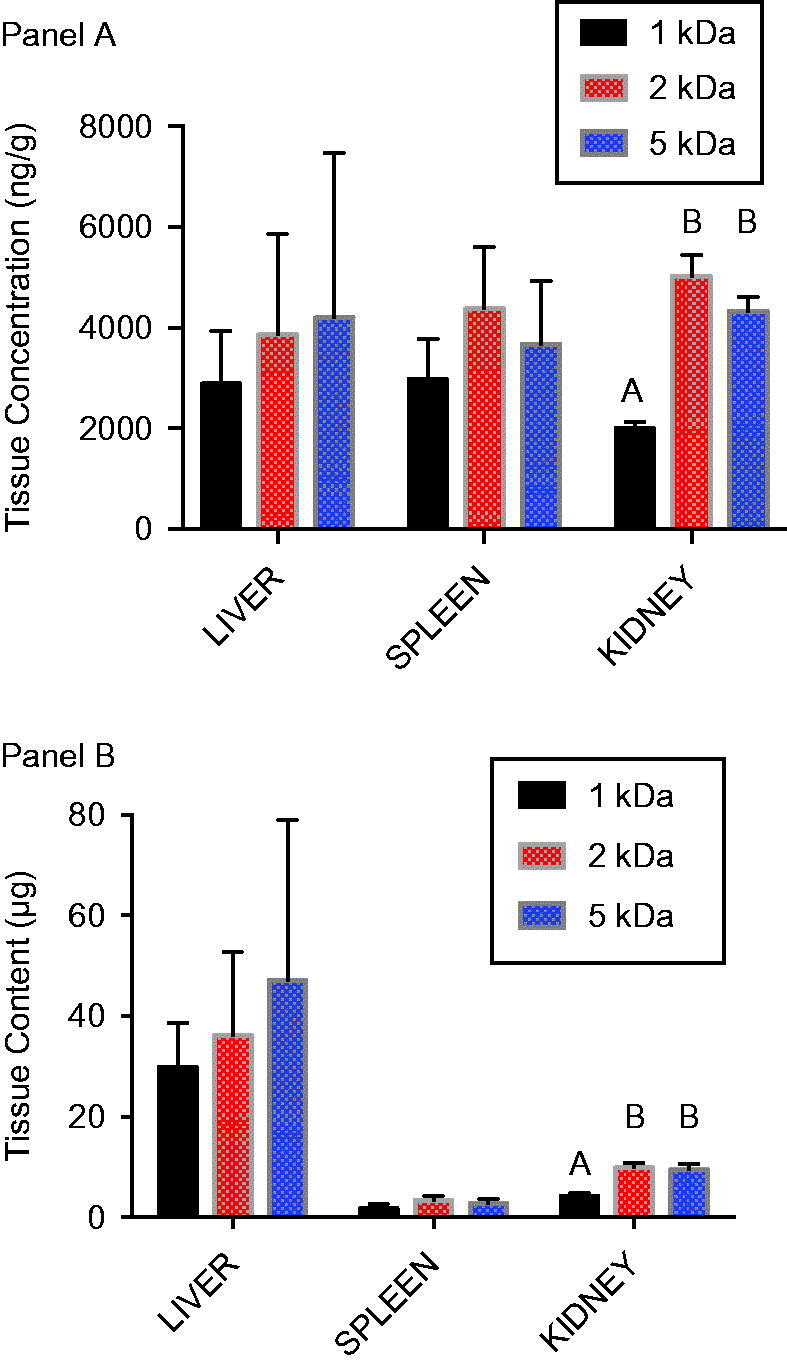
Tissue distribution of 1, 2 and 5 kDa PEG-coated 5 nm AuNPs following intravenous administration. Gold content was measured in liver, spleen, and kidney at sacrifice 24 h after an intravenous dose (0.8 mg/kg) of either 1, 2 or 5 kDa PEG-coated 5 nm AuNPs. A) Gold concentration in tissues. B) Mass of gold in the tissue based upon tissue concentration and total weight of the organ. Results are presented as mean ± SD, *N* = 5. Bars with letter superscripts (A or B) denote significant differences among PEG-coated particle types; bars with different letter superscripts are significantly different *p* < 0.05.

Distribution of AuNPs to liver, spleen, and kidneys was assessed 24 h after a single intravenous dose (0.8 mg/kg). PEG-coated AuNPS were found in roughly similar concentrations in liver, spleen, and kidney (Figure 4(A)). Statistically significant differences were noted in kidney AuNP concentrations; however, when compared with AuNP concentrations in other tissues, the kidney concentrations were generally within the range of concentrations observed in liver and spleen. Because of the larger size of the liver, total mass of AuNP in the liver was substantially higher than spleen or kidney (Figure 4(B)).

**Figure 5.**
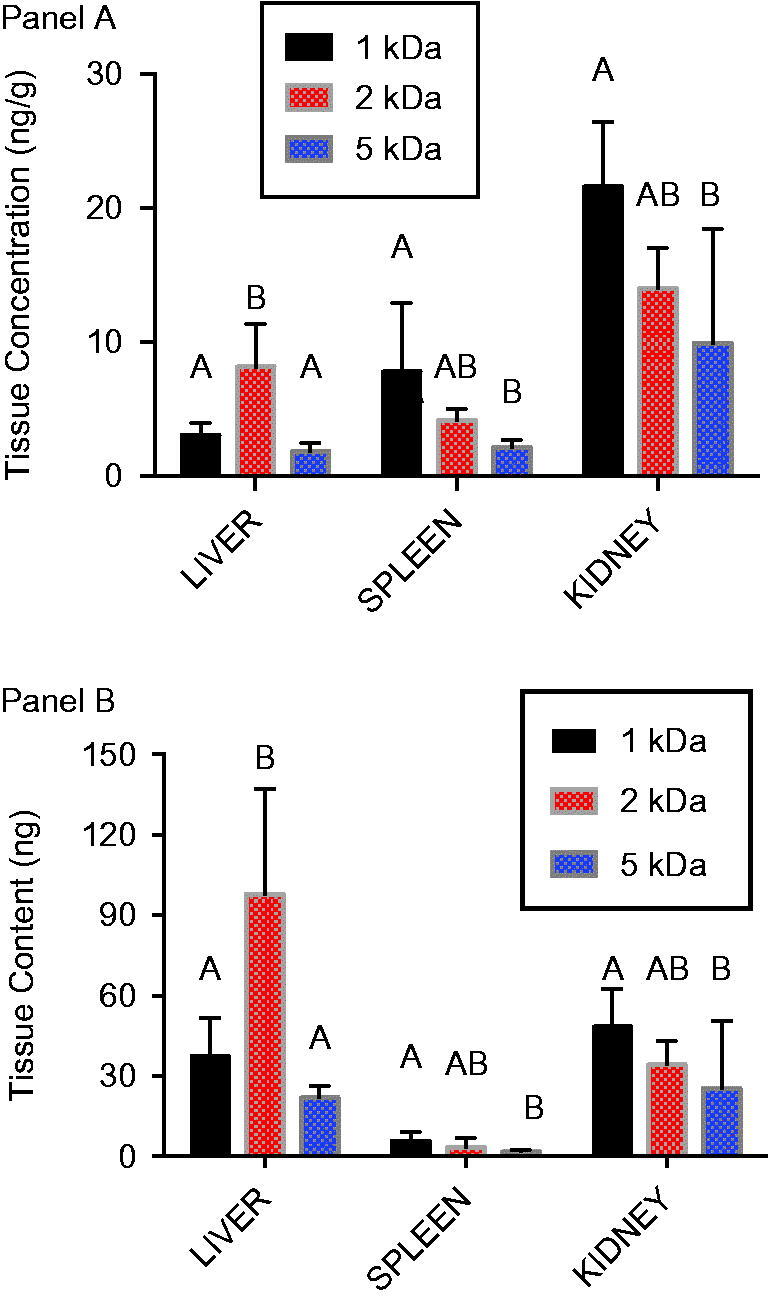
Tissue distribution of 1, 2 and 5 kDa PEG-coated 5 nm AuNPs following oral administration. Gold content was measured in liver, spleen, and kidney at sacrifice 48 h after an oral dose (8.0 mg/kg) of either 1, 2 or 5 kDa PEG-coated 5 nm AuNPs. A) Gold concentration in tissues. B) Mass of gold in the tissue based upon tissue concentration and total weight of the organ. Results are presented as mean ± SD, *N* = 5. Bars with letter superscripts (A, B or AB) denote significant differences among PEG-coated particle types; bars with different letter superscripts are significantly different *p* < 0.05.

Based upon the blood concentration over time profiles after oral administration (Figure 3), it was hypothesized that tissue concentrations would show a declining trend with increasing PEG coating size. This was the case for spleen and kidney, but not liver (Figure 5(A)). In kidney and spleen, significantly higher AuNP concentrations were observed following 1 kDa PEG AuNP administration compared with administration of 5 kDa PEG-coated particles, with 2 kDa PEG-coated particles present in intermediate concentrations. For liver, 1 and 5 kDa PEG-coated AuNP concentrations were similar, while concentrations for the 2 kDa PEG-coated particles were significantly higher. While the liver clearly held the largest mass of particles after intravenous administration (Figure 4(B)), higher concentrations in kidney [relative to liver] after oral administration resulted in more comparable overall tissue burden of AuNPs (Figure 5(B)).

## Discussion

The utility of metallic nanoparticles as drug delivery devices for the oral route depends in large part on their bioavailability. To date, most assessments of the oral bioavailability of metallic nanoparticles have relied upon measurement of nanoparticles in urine, feces, or tissues after one or more doses. Use of measurement of the percent dose excreted in urine and feces to estimate bioavailability can be confounded if there is significant biliary excretion, which has been demonstrated for several metallic nanoparticles in mice and rats (Lee et al., 2013; Zhang et al., 2016). Examples of nanoparticles for which there is evidence of biliary excretion include gold, silver, zinc oxide, manganese oxide and iron nanoparticles, as well as silica nanoparticles. Often, estimates of the extent of biliary excretion are made based upon the percent of an intravenous dose excreted in feces. Using this approach, observations regarding the extent of biliary excretion vary widely among different nanomaterials, but extend up to nearly 100% (Zhang et al., 2016). If biliary excretion of a nanoparticle is substantial, this can result in an underestimation of oral bioavailability from urinary and fecal measurements. Also, there is evidence that ingested nanoparticles can become trapped within the gastrointestinal tract, neither absorbed systemically nor excreted in feces for a month or more after an oral dose (Hinkley et al., 2015). This too can lead to errors in bioavailability assessment from fecal measurements, at least in theory.

There are very few studies assessing the oral bioavailability of metallic nanoparticles using the classical method of comparing the AUC in blood after oral and intravenous doses. Kim et al. (2016) used this approach successfully to compare the oral bioavailability of nano versus bulk scale silica, titanium dioxide and zinc oxide in rats. Baek et al. (2012) measured the AUCs of zinc in blood following repeated oral doses to estimate bioavailability of zinc oxide nanoparticles, but did not administer the particles intravenously for comparison. Instead, the absorbed dose was estimated from the oral AUC. Park et al. (2011) examined the bioavailability of citrate-coated silver nanoparticles in rats after intravenous and oral doses of 1 and 10 mg/kg, including an attempt to use intravenous and oral AUC comparisons. This approach was unsuccessful because blood concentrations were too low to derive an oral AUC following the 1 mg/kg dose, and blood concentrations following the 10 mg/kg intravenous dose did not decrease during the 96 h observation period precluding estimation of an AUC.

The study presented here used blood AUCs after intravenous and oral doses to compare the oral bioavailabilities of AuNPs coated with PEGs of different sizes. Using this approach, an inverse relationship was observed between PEG coating size and oral bioavailability, with AuNPs coated with 1 kDa PEG having much greater oral absorption than AuNPs coated with either 2 or 5 kDa PEG. Donovan et al. (1990) found increasing gastrointestinal absorption of PEG molecules with decreasing size beginning with sizes around 1 kDa. The observations here are also consistent with the findings of Smith et al. (2013), who examined the oral absorption of 2 nm AuNPs coated with smaller sizes of PEG (down to about 400 Da) (Smith et al., 2013). They observed comparatively higher absorption of the particles coated with the smallest PEG based upon measurement of blood and tissues at discrete time points (1, 8 and 24 h after the dose). Urine collection during the experiment was incomplete, but based upon the mass of AuNPs collected in urine and the administered dose, they estimated the average absorbed dose for the 400 Da PEG-coated AuNPs to be at a minimum 5%. The authors noted that this is orders of magnitude higher than oral bioavailability estimates for AuNPs in other studies in the literature and their AuNPs coated with larger PEGs, and it is also much higher than the oral bioavailabilities seen in our study with larger PEG molecule coatings. The prospect of substantial oral bioavailability of AuNPs coated with small (e.g. 400–600 Da) PEG is promising from the standpoint of drug delivery. It will be important confirm these using a more definitive, quantitative approach to assessing bioavailability such as the blood AUC approach used here.

After an intravenous dose of the three PEG-coated AuNP types, gold content in the liver was much higher than in the spleen or kidney (Figure 4(B)). This is consistent with observations of Lipka et al. (2010), who found nanoparticle content of liver + spleen to be higher than kidneys after intravenous administration of radiolabeled, PEG-coated AuNPs. However, concentration measurements in the study here show similar concentrations of gold from each particle type in liver, spleen, and kidney (Figure 4(B)), and the differences in tissue content are essentially a function of organ size. After oral administration, kidney concentrations of AuNPs were generally higher than liver or spleen (Figure 5(B)), which is also consistent with observations by Smith et al. (2013) with different PEG-coated AuNPs and by Hillyer & Albrecht (2001) in mice given colloidal gold of different sizes orally.

Inferences regarding oral bioavailability of nanoparticles are commonly made using tissue concentration measurements such as these. In general, quantitative measurement of oral bioavailability using tissue measurements at a given time point is subject to error unless the material has been dosed to steady state, which is seldom demonstrated in studies of nanomaterials. Further, concentrations in individual tissues are a function not only of the absorbed dose, but also the extent of tissue uptake. This can confound the use of tissue concentration data to assess bioavailability, especially the relative bioavailability of materials that may have different affinities. This problem is illustrated by tissue concentration data in the study here. The rank order of gold concentrations in spleen and kidney after oral administration of PEG-coated AuNPs (1 > 2 > 5 kDa PEG-coated particles; Figure 5) is qualitatively similar to the rank order of their AUCs in blood (Table 1), although the magnitude of the differences among the particles are not the same. The liver presents a very different picture, however, with concentrations of the 2 kDa PEG-coated particles significantly higher than the other particles. Looking only at liver tissue data, a conclusion might be reached that uptake of 2 kDa PEG-coated particles was greater than that of the 1 kDa or 5 kDa particles, which would be in error. Conclusions regarding comparative bioavailability that rely solely on tissue data must be therefore be made with caution.

## Conclusions

Quantitative evaluation of the oral bioavailability of metallic nanoparticles is a critical aspect of their assessment as potential tools for drug delivery. To date, most studies of nanoparticle uptake following oral administration have used approaches that are at best semi-quantitative, and there is a need to apply more rigorous methods. The study presented here with PEG-coated AuNPs demonstrates the utility of the classical bioavailability approach of comparing blood concentration profiles over time following oral and intravenous administration to quantify oral bioavailability of metallic nanoparticles. The oral bioavailabilities of the 1, 2 and 5 kDa PEG-coated AuNPs were all found to be very low. Evaluation of AuNPs with sub-1 kDa PEG coating is a logical next step using definitive bioavailability assessment methods
